# Injury Prediction and Risk Modelling in Team Sports Using Artificial Intelligence and Sensor-Based Monitoring: A Scoping Review

**DOI:** 10.3390/jfmk11020204

**Published:** 2026-05-22

**Authors:** Michail Tsenos, Christos Kokkotis, Dimitrios Draganidis, Nikos Alibertis, Dimitrios Pantazis, Panagiotis Tsimeas, Athanasios Poulios, Nikolaos Zaras, Paraskevi Malliou, Ilias Tsaousidis, Maria Michalopoulou, Dimitris Tsakalidis, Alexandra Avloniti, Ioannis G. Fatouros, Athanasios Chatzinikolaou

**Affiliations:** 1 Department of Informatics, Athens University of Economics and Business, 10434 Athens, Greece; tsemike@aueb.gr; 2 Department of Occupational, School of Physical Education, Sport Science and Occupational Therapy, Democritus University of Thrace, 69100 Komotini, Greece; ckokkoti@ot.duth.gr; 3 Department of Physical Education and Sport Science, School of Physical Education, Sport Science and Dietetics, University of Thessaly, 43100 Trikala, Greece; ddraganidis@uth.gr (D.D.); ptsimeas@uth.gr (P.T.); apoulios@uth.gr (A.P.); itsaousidis@uth.gr (I.T.); ifatouros@uth.gr (I.G.F.); 4 Novelcore, 10436 Athens, Greece; alimpertis@novelcore.eu (N.A.); tsakalidis@novelcore.eu (D.T.); 5 Department of Physical Education and Sport Science, School of Physical Education, Sport Science and Occupational Therapy, Democritus University of Thrace, 69100 Komotini, Greece; dpantazi@phyed.duth.gr (D.P.); nzaras@phyed.duth.gr (N.Z.); vivianmalliou@gmail.com (P.M.); michal@phyed.duth.gr (M.M.); alavloni@phyed.duth.gr (A.A.)

**Keywords:** machine learning, injury risk modelling, wearable technology, athlete monitoring, training load, sports injuries

## Abstract

Sports-related injuries remain a major challenge in team sports, with important consequences for athlete health, performance, and team success. Recent advances in artificial intelligence (AI) and sensor-based monitoring technologies have enabled the integration of large volumes of training, competition, and physiological data to support injury prediction and risk modelling. However, the literature is characterised by substantial methodological diversity, limiting the ability to draw consistent conclusions. Hence, this scoping review aimed to map the existing evidence on the use of AI and sensor-based monitoring technologies for injury prediction and risk modelling in team sports, and to identify key methodological trends and research gaps. The scoping review was conducted in accordance with the PRISMA-ScR guidelines. Systematic searches were performed in PubMed and Scopus. Eligible studies included team-sport athletes and applied AI or machine learning approaches to predict injury occurrence, injury risk, or related outcomes using data derived from wearable or monitoring systems. Data were charted on study characteristics, sports and competition level, data sources, modelling techniques, validation strategies, and performance metrics. The database search yielded 123 records (PubMed: *n* = 37; Scopus: *n* = 86). After screening and eligibility assessment, 11 studies met the inclusion criteria. Most studies focused on football and rugby and relied primarily on wearable-derived data, particularly GPS and inertial sensor outputs. Common predictors included external workload variables, training exposure, previous injury history, and, in some studies, wellness or physiological markers. A wide range of models was reported, including logistic regression, decision trees, random forests, support vector machines, and neural networks. Validation strategies and reported performance varied markedly, and external validation was rarely undertaken. Across the included studies, injury risk was most consistently associated with external workload metrics, previous injury history, and internal or physiological indicators of recovery and readiness. However, current models remain limited by heterogeneous methodologies, single-team datasets, and the lack of external validation. Future research should emphasise multimodal data integration and multi-centre validation to develop reliable, interpretable, and practically applicable AI-based injury prediction systems.

## 1. Introduction

Sports-related injuries remain a major challenge in team sports, with substantial implications for athlete health, performance, and career longevity, as well as for the economic and organisational sustainability of sporting programmes. Injury epidemiology has traditionally focused on quantifying injury incidence, prevalence, and severity, and on identifying risk factors associated with injury occurrence [1,2]. These efforts provide the foundation for evidence-based injury prevention strategies and guide medical and training decisions in applied settings. In addition to descriptive epidemiology, considerable efforts have been made to estimate and model injury risk, particularly through the monitoring of training load and its relationship with injury occurrence. Early approaches focused on workload-based models, including cumulative load, training monotony, and strain, as well as the acute-to-chronic workload ratio (ACWR), which has been widely used to assess fluctuations in training demand and their association with injury risk [3,4,5]. A large body of evidence has demonstrated a consistent association between training load and injury risk across different athletic populations [6]. Furthermore, the concept of the training–injury prevention paradox suggests that appropriately high chronic workloads may have a protective effect against injury, whereas rapid spikes in load increase injury risk [5]. Empirical studies using ACWR models have also shown that sudden increases in workload are associated with significantly higher injury likelihood [7]. Although such models have contributed to understanding load–injury relationships, their predictive capacity remains limited when applied at the individual level, highlighting the complexity and multifactorial nature of injury mechanisms.

The rapid development of wearable technologies and athlete monitoring systems has enabled the continuous collection of high-resolution data across multiple domains. External load is commonly quantified using global positioning system (GPS) devices and inertial measurement units (IMUs), capturing variables such as distance covered, velocity profiles, and acceleration–deceleration patterns [8,9]. In parallel, internal load can be assessed through physiological and perceptual measures, including heart rate responses, session rating of perceived exertion (sRPE), and subjective wellness indicators [10,11]. Monitoring both internal and external load has been widely recommended to better understand athlete responses and reduce injury risk [12]. Practical frameworks have also emphasised the importance of combining physiological and biomechanical load pathways to understand adaptation and injury mechanisms [13]. Additionally, tracking systems have been shown to support training planning and injury risk management through objective quantification of external load [14]. The integration of these data sources allows for a more comprehensive representation of the athlete’s response to training, facilitating the translation of external workload into internal physiological stress. With the widespread adoption of athlete monitoring systems, including wearable sensors and digital performance tracking technologies, injury surveillance has progressively shifted from descriptive reporting toward more data-driven and predictive approaches.

External load refers to the physical work completed by the athlete and is commonly quantified through metrics such as total distance covered, high-speed running, sprint distance, accelerations, and decelerations derived from GPSs, local positioning systems (LPSs) and inertial sensor systems. On the other hand, internal load reflects the individual physiological and psychological response to the imposed workload and is typically assessed using measures such as heart rate, sRPE, wellness questionnaires, and fatigue-related indicators. The distinction between internal and external load is important because athletes may respond differently to similar external demands depending on fitness level, recovery status, and contextual factors.

Early investigations into injury risk in sport have primarily relied on traditional statistical methods to examine associations between workload, physical characteristics, and injury outcomes. Techniques such as linear and logistic regression, survival analysis, and correlation-based models have been widely applied to estimate injury risk and to explore potential predictors at the population level [15]. Traditional monitoring approaches have also incorporated physiological and perceptual markers such as heart rate, training impulse (TRIMP), and subjective fatigue measures to evaluate training stress and injury risk [11]. Heart rate monitoring has been extensively used as a valid indicator of internal load and physiological stress in team sports [16]. More broadly, training load monitoring frameworks combining internal and external metrics have been proposed as essential tools for optimising performance and minimising injury risk [17]. Although these approaches have generated important insights into injury mechanisms and training–injury relationships, they are inherently limited in their capacity to handle high-dimensional data, complex interactions, and non-linear patterns [18]. Furthermore, traditional statistical models often assume stable and homogeneous relationships across individuals, which may not adequately reflect the dynamic and multifactorial nature of injury risk in elite and sub-elite sport contexts [3].

In the modern era of sport science, the rapid expansion of data availability and the development of advanced analytical techniques have transformed how athlete performance and injury risk are assessed. More recently, machine learning (ML) has emerged as a promising alternative for injury prediction and risk modelling. ML approaches differ from traditional statistical techniques because they are designed to identify complex, non-linear, and multidimensional relationships within large datasets without requiring predefined assumptions regarding variable interactions. Such approaches are particularly relevant in sports injury prediction, where injury occurrence is influenced by multiple interacting physiological, biomechanical, historical, and contextual factors. Specifically, ML approaches are well suited to analysing large and heterogeneous datasets derived from wearable devices, training logs, and physiological monitoring systems, and may better capture interactions among workload variables, contextual factors, and individual responses [19,20,21]. Alongside these developments, explainable artificial intelligence (XAI) techniques have been introduced to improve the interpretability of complex predictive models. By providing information on feature importance and decision pathways, XAI methods address concerns related to the “black box” nature of many ML algorithms and support transparency and trust in applied environments [22,23]. In sports medicine and performance settings, interpretability is particularly important to facilitate communication between analysts, clinicians, and coaching staff, and to enable the translation of model outputs into practical and ethical decision-making.

Despite growing interest in AI-based injury prediction, the existing literature remains methodologically heterogeneous and fragmented [24]. Studies differ considerably in terms of the sports examined, the type and granularity of sensor-derived data, feature engineering strategies, modelling techniques, validation procedures, and performance metrics. In addition, limited attention has been devoted to external validation and to the clinical interpretability of model outputs, which constrains the generalisability and real-world applicability of many proposed models. To date, no synthesis has comprehensively mapped how AΙ and sensor-based monitoring technologies have been applied to injury prediction and risk modelling across team sports. Therefore, the aim of this scoping review is to systematically map the literature on the use of AI and sensor-based monitoring technologies for injury prediction and injury-related risk modelling in team sports, primarily focusing on studies examining time-loss, non-contact, or workload-related injury outcomes. Specifically, this review aims to describe the characteristics of the included studies, the data sources and modelling approaches employed, and the strategies used for model validation and interpretation, while identifying key methodological limitations and priorities for future research.

## 2. Materials and Methods

This scoping review was conducted in accordance with the Preferred Reporting Items for Systematic Reviews and Meta-Analyses extension for Scoping Reviews (PRISMA-ScR) 22-item checklist to ensure a rigorous, consistent, and transparent review process [25,26,27,28]. By adhering to the PRISMA-ScR framework, this study sought to maintain high methodological standards, facilitate reproducibility, and enhance the reliability and validity of the qualitative synthesis. The protocol for this scoping review was prospectively registered on the Open Science Framework (OSF; https://osf.io/xnr7q, accessed on 30 January 2026) to enhance methodological transparency and reproducibility.

### 2.1. Literature Searches

A systematic literature search was performed using the PubMed and Scopus databases. These databases were selected to ensure comprehensive coverage of biomedical, sports science, and technology-oriented research. PubMed and Scopus were selected because together they provide extensive coverage of biomedical, sports science, engineering, and interdisciplinary research relevant to athlete monitoring, wearable technologies, and AI-based modelling. In addition, Scopus includes broader indexing of sports technology and data science literature, while PubMed provides strong coverage of sports medicine and health-related research. Searches were conducted on 18 January 2026. The search strategy combined keywords and phrases related to team sports, sports injuries, injury prediction and risk modelling, artificial intelligence, and sensor-based monitoring technologies. Searches were applied to the Title and Abstract fields in each database and included terms associated with AI and ML (e.g., “artificial intelligence”, “machine learning”, “deep learning”), injury-related outcomes (e.g., “injury prediction”, “risk modelling”, “prognosis”), team sports (e.g., “football”, “rugby”, “basketball”, “hockey”), and wearable or monitoring technologies (e.g., “global positioning system”, “inertial measurement units”, “wearable sensors”, “training load”). The full search strategies for each database are provided in the Supplementary Materials File S1.

### 2.2. Eligibility Criteria

#### 2.2.1. Inclusion Criteria

Only peer-reviewed journal articles were considered to ensure scientific quality and reliability. The review focused on studies published between 1 January 2014 and 18 January 2026 in order to capture contemporary developments in AI and athlete monitoring technologies. The 2014 starting date was selected because it coincides with the broader adoption of wearable athlete monitoring technologies and the increasing application of contemporary machine learning methods within sports performance and injury research. Eligible studies were required to involve athletes participating in team sports and to apply AI or ML approaches to predict injury occurrence, injury risk, or related injury outcomes. Studies were also required to use data derived from wearable devices or athlete monitoring systems, such as global positioning systems (GPSs), inertial measurement units (IMUs), physiological sensors, or workload monitoring platforms.

#### 2.2.2. Exclusion Criteria

Studies were excluded if they were published before 2014, were not written in English, or were not peer-reviewed journal articles. Additional exclusions included conference proceedings, books, editorials, commentaries, and review articles. Studies were also excluded if they did not address injury-related outcomes, did not apply artificial intelligence or machine learning methods, focused exclusively on individual sports or non-athlete populations, or examined rehabilitation or return-to-play processes without a predictive modelling component. Articles with inaccessible full texts were also excluded to ensure complete evaluation. For additional reporting transparency, the eligibility criteria were also structured according to an adapted PICOS framework provided in Appendix A (Table A1).

### 2.3. Data Extraction

The study selection process was conducted independently by two reviewers (M.T and C.K.), who screened titles, abstracts, and full texts for relevance according to the predefined eligibility criteria. All retrieved records were initially compiled into a spreadsheet, and duplicate entries were removed. Title and abstract screening was performed first, followed by full-text assessment of potentially eligible articles. Disagreements at any stage of the screening process were resolved through discussion and consensus.

Following study selection, data extraction was carried out independently by the same two reviewers to ensure consistency and accuracy. A standardised data charting form, which was piloted by the review team prior to full extraction, was used to capture key variables across studies. Extracted information included publication characteristics, sport and competition level, sample size and participant characteristics, data sources and monitoring technologies, input variables, AI or ML models applied, validation strategies, and reported performance outcomes. A descriptive synthesis approach was employed to summarise and organise the charted data, allowing for identification of patterns and thematic trends across studies in terms of data sources, modelling approaches, and methodological quality. This structured process ensured that the final set of included studies was both relevant to the research question and methodologically robust.

## 3. Results

**Figure 1:**
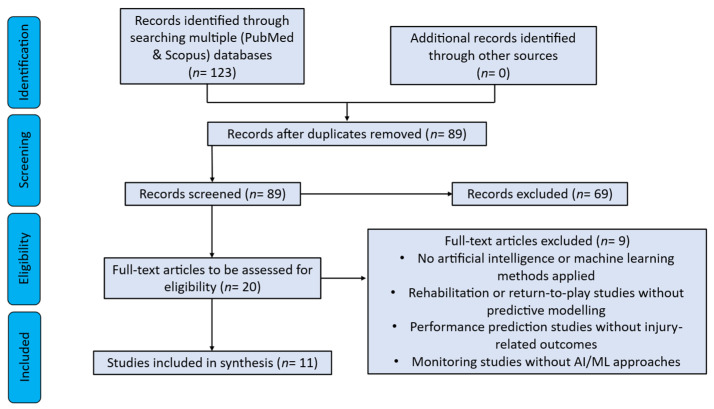
Workflow diagram of the screening methodology according to PRISMA-ScR guidelines.

The initial database search yielded a total of 123 records, including 37 articles from PubMed and 86 articles from Scopus. After removal of duplicate records, the remaining studies were screened based on their titles and abstracts. Articles that did not address injury prediction or risk modelling in team sports using artificial intelligence and monitoring technologies were excluded. Full-text assessment of potentially eligible studies was subsequently performed. Following this screening and eligibility process, 11 studies met the predefined inclusion criteria and were included in the qualitative synthesis. The study selection process is illustrated in Figure 1 using a PRISMA-ScR flow diagram.

### 3.1. Characteristics of Included Studies

The 11 included studies were published between 2014 and 2026 and were conducted predominantly in Europe and Australia, with a smaller number originating from North America. Most investigations focused on professional or elite-level athletes, while a smaller proportion included semi-professional or youth populations.

Football was the most frequently investigated sport, followed by rugby, American football, and Australian football. The duration of data collection varied across studies, ranging from single-season monitoring of individual teams to multi-season datasets including several squads. Sample sizes also differed considerably, reflecting variations in team size, observation periods, and data availability.

Injury outcomes were defined heterogeneously. Most studies focused on time-loss injuries, whereas others adopted broader definitions based on physical complaints or medical diagnosis. The majority of the studies employed retrospective designs using historical training and injury records, while only a limited number implemented prospective or temporally structured prediction approaches. Among the included studies, most adopted retrospective observational designs (*n* = 8), while only a limited number used temporally separated or prospective prediction frameworks (*n* = 3). Sample sizes ranged from 18 to 133 athletes, and monitoring periods varied from single-season datasets to multi-season longitudinal observations spanning up to four seasons. Details of each included study are presented in Table 1.

### 3.2. Data Sources and Monitoring Technologies

All included studies utilised data derived from athlete monitoring systems. Wearable technologies constituted the primary source of input data, particularly GPS devices and IMUs. External workload variables were consistently reported and included total distance covered, high-speed running distance, sprint distance, and counts of accelerations and decelerations. Several studies also incorporated cumulative workload measures, rolling averages, and workload ratios to represent short-term and long-term training exposure.

In addition to external workload metrics, a subset of studies integrated internal load indicators, such as heart rate-based variables, ratings of perceived exertion, and subjective wellness or fatigue scores. Historical injury information was commonly used as an input variable, alongside contextual factors including playing position, match exposure, training frequency, and competition density. Some studies also incorporated calendar-related variables, such as days between matches or training sessions.

Feature construction and temporal aggregation strategies varied across investigations. Differences were observed in the length of aggregation windows, the frequency of feature updates, and the use of raw versus derived variables. Preprocessing approaches, including data normalisation and handling of missing values, were not consistently described across studies.

### 3.3. Modelling Approaches and Predictive Outcomes

A wide range of AI and ML techniques were applied across the included studies. Commonly reported models included logistic regression, decision trees, random forests, support vector machines, and artificial neural networks. Several investigations compared multiple algorithms within the same dataset, whereas others focused on a single modelling approach. Tree-based and ensemble methods were among the most frequently employed techniques.

Validation strategies varied across studies. Most relied on internal validation procedures, such as k-fold cross-validation or split-sample testing, whereas external validation using independent datasets was rarely reported. Only a small number of studies adopted temporally separated training and testing periods to reflect prospective prediction scenarios.

Performance was assessed using a range of metrics, most commonly accuracy, sensitivity, specificity, and the area under the receiver operating characteristic curve (AUC). Some studies also reported precision, recall, or F1-scores to account for class imbalance. A limited number of studies reported the use of class imbalance handling techniques or analyses of model explainability.

Considerable methodological heterogeneity was observed not only in modelling approaches but also in feature engineering procedures, injury definitions, aggregation windows, and validation frameworks, limiting direct comparison across studies and reducing reproducibility.

### 3.4. Study-by-Study Summary of Application Domains

Across the 11 included studies, the vast majority concentrated on injury prediction in football. Martins et al. used longitudinal monitoring data to model muscle injury risk, with tree-based ensemble methods performing best and both internal load (RPE) and external workload variables, including total distance, accelerations, decelerations, high-speed running, and time spent in high-intensity zones, identified as the most influential features [29]. Similarly, Saberisani et al. [30] and Freitas et al. [31] applied machine learning models to GPS-derived workload data, reporting the best performance with ensemble or support vector machine approaches. In these studies, distances covered at moderate and high speeds, decelerations, player load, velocity, acceleration, session type, and playing position were among the most important predictors. Tsilimigkras et al. also used training load data in soccer, with gradient-boosted models highlighting both acute load deviations, such as sprint counts, heart rate-based load, and time spent at 90–100% of maximal heart rate, and cumulative load variables, including total distance, high-speed running, and sprint distance [32].

Two studies by Rossi and colleagues explored different modelling strategies. In their earlier work, Rossi et al. used GPS-derived training data and identified three key predictors, time since previous injury (PI), high-speed running variation (dHSR), and total distance monotony (dTOT), through recursive feature elimination, with a decision tree providing the best performance [19]. In a later study, Rossi et al. incorporated blood-derived biomarkers alongside monitoring data, showing improved predictive performance when hematological and hormonal variables such as hematocrit, hemoglobin, red blood cell count, ferritin, and testosterone were included alongside external workload metrics [33].

In rugby contexts, Duffuler et al. applied a recurrent neural network to model readiness and injury risk across a season, capturing sequential workload patterns [34]. Key predictors included high-intensity running distances in preceding days and player age, indicating the importance of both recent workload exposure and individual characteristics. Ren et al. used GPS-derived metrics and machine learning models to predict injury risk in professional rugby players, with random forest models achieving the best results [35]. The most influential predictors varied by playing position and included monotony of sprint and very high-speed running, cumulative acceleration loads, high-speed running distance, and high-intensity accelerations and decelerations.

Matas-Bustos et al. focused on feature engineering rather than a specific sport context, proposing advanced methods for calculating acute-to-chronic workload ratios [36]. Their gradient-boosted models demonstrated improved injury forecasting when variables such as total distance, speed, high-intensity efforts, accelerations, decelerations, impacts, player load, and workload changes were incorporated.

Outside of association football, Lyubovsky et al. examined injury prediction in American football using GPS, training load, and survey-based wellness data [37]. Their models showed moderate predictive performance, with subjective wellness indicators and injury history, particularly previous injuries, perceived soreness, and recovery status, emerging as the most influential predictors, while many GPS-derived variables showed limited contribution. In Australian football, Carey et al. applied multivariate predictive models based on training load data [38]. Although model performance was modest, cumulative load, acute and chronic workload, ACWR, monotony, and strain were identified as key variables, with improved performance observed when modelling specific injury types.

Across all studies, considerable variability was observed in prediction targets, input variables, modelling techniques, and validation strategies, as detailed in Table 1.

**Table 1:** Characteristics of included studies.

Author	Application Domain	Monitoring Technology	Measurements	Data & Subjects	Feature Selection/Dimensionality Reduction	Machine Learning Models	Validation Strategy	Explainability	Results	Key Predictors /Important Variables
Martins et al. (2025) [29]	Injury risk prediction in football	Wearable GPS 10 Hz GPS unit, EVO (Catapult, Melbourne, Australia), and a 10 Hz GPS unit, Apex pro series (STATSports, Newry, Northern Ireland, UK); heart rate monitors, performance tracking software, internal/external load monitoring	External load (GPS), internal load (HR, RPE), anthropometry, training/match performance metrics, previous injury history Injury outcome/definition: muscle injury	Training sessions and official matches, four consecutive sporting seasons, professional football club competing in the Portuguese professional football leagues (N = 96 males)	NA	IBk, K-Star, simple logistic, logistic classifier, MLP classifier, RF, RBF classifier, SMO	10-fold CV	NA	K-Star classifier with AUC-PR of 83% and a balanced accuracy of 72% from the four-week window. MLP classifier in the two-week window before each injury occurred demonstrated an F1-score of 71% and a balanced accuracy of 72%	RPE, total distance, accelerations/decelerations, high-speed running, high-intensity zones
Duffuler et al. (2025) [34]	Injury prevention and readiness prediction in rugby	GPS device used was the Vector V7 from Catapult, which provides information at frequencies of 10 and 100 Hz	Training time, distance above specific speeds, number of accelerations/decelerations, total number of accelerations over 5G Injury outcome/definition: intrinsic injuries	Screening data DFT, DROM, AKE, Schober test/contextual data/environmental data/status data/Injury data. Professional club-level male athletes (N = 72)	Conv1D layers	LR, DT RF, RNN, LSTM, convolutional layers with RNN and XGBoost	Validation set	LIME	Conv1D + LSTM-3 accuracy of 92.9% and F1-score of 0.83	High-intensity running (preceding days), player age
Ren et al. (2025) [35]	Injury prevention in rugby	GPS (specifically Catapult Vector X7 sensors) and specialised GPS software (Openfield Console 3.7)	17 GPS-derived workload metrics, including total distance, medium/high/very high-speed running distances, sprint distance (SR), repeated high-intensity effort (RHIE), contact involvement, and various acceleration/deceleration zones Injury outcome/definition: non-contact injuries	Professional male players (N = 63) from a team in the French Pro D2 division. Training and matches from three seasons	Pearson correlation (>0.7 were removed). GBDT with an importance score threshold > 0.01	LR, NB, SVM, RF, and XGBoost	70% training (internal 10-fold CV), 30% testing	Weighted feature importance (WFI)	RF performed best for forwards, with XGBoost excelling in the tight five and SVM in the back row. NB demonstrated the highest performance for backs overall, with average F1-scores of up to 0.66 (±0.14).	HSR/VHSR/SR metrics, acceleration/deceleration zones, workload monotony, cumulative load (position-specific)
Matas-Bustos et al. (2025) [36]	Injury prevention in football	EPTS	HRVM and GPS. External workload variables including distance, speed, acceleration, deceleration, step balance, player load, jump count, and impact forces. It also includes variables describing the distribution of speed and acceleration across ranges. Injury outcome/definition: muscle injuries	23 male professional athletes; data include both training sessions (on-pitch) and official matches. Spanish Primera División (LaLiga)	PCA	LDA LR NB KNN SVM CART, RF, MLP	10-Repeated-stratified-2-fold cross-validation	NA	The employed models (with FWF cumulative and temporary variations) achieved a best ROC-AUC of 0.7707 [95% CI: 0.7407–0.8094] and a best PR-AUC of 0.4509 [95% CI: 0.4168–0.4864].	Distance, speed, high-intensity efforts, accelerations, decelerations, player load, ACWR, workload changes
Saberisani et al. (2025) [30]	Injury prevention in football	GPS-based wearable sensors (10 Hz ST2)	External load metrics, including total distance covered, average distance covered, distances at high and moderate speeds, total distance load, accelerations, and decelerations Injury outcome/definition: Weeks were labelled as “injury” when a player sustained a collision or non-collision injury causing absence from at least one training session or match. All injuries meeting these criteria and recorded by the team physician were included according to the Fuller et al. classification [39].	Data were collected from both training sessions (214) and competition sessions (34) during a full league season from 25 males with goalkeepers in the Persian Gulf Pro League in Iran.	NA	DT	80% training, 20% testing	NA	AUC > 0.95	Moderate/high-speed distance, decelerations
Freitas et al. (2025) [31]	Injury prevention in football	GPS receivers from Catapult (GPSports EVO), sampling frequency of 10 Hz	Catapult system to derive a total of 1379 parameters reduced to 424 GPS metrics for this study, like distance, velocity, acceleration Injury outcome/definition: traumatic and overload injuries	Data per exercise and session were collected by the GPS receivers and extracted using Catapult’s GPSports Cloud for 34 male players.	mRMR	SVM, FNN, AdaBoost	Stratified CV	NA	SVM 74.22% accuracy	Player load, velocity, acceleration, session type, player position
Tsilimigkras et al. (2024) [32]	Injury prevention in football	Wearable GPS devices (10 Hz) integrated with 3D accelerometers, gyroscopes, and heart rate recording belts/vests (Polar Team Pro system)	External: number of sprints, number of accelerations, number of decelerations, and total distance covered. Internal: HR data and derivatives such as time in HR zones Injury outcome/definition: all injuries	Professional male players (n = 25 with goalkeepers) in Asteras Tripolis. Recorded during all training sessions and official matches over a 4-year period (Greek Super League seasons 2021–2024)	RFE-correlation bias reduction (CBR)	SVM-RBF compared against SVM, k-NN, LDA, and RF	LOOCV/1000 permutation tests under the same LOOCV	NA	Accuracy of 0.78 (*p* < 0.01, 1000 permutations), sensitivity = 0.73, specificity = 0.85	Sprint count, HR-based load, time at high HR, total distance, HSR, sprint distance
Rossi et al. (2018) [19]	Injury prevention in football	10 Hz GPS devices integrated with a 100 Hz 3-D accelerometer, a 3D gyroscope, and a 3D digital compass (STATSports Viper) software package Viper version 2.1	External loads, speed, accel, decel, distance at metabolic power, age, play time, BMI Injury outcome/definition: A non-contact injury was defined as any tissue damage sustained by a player that resulted in absence from physical activity for at least one day following the onset of the injury.	Italian professional, a total of 931 individual training sessions were recorded during the 23 weeks from 26 male players	EWMA, ACWR, MSWR. RFE with CV	DT, RF and a Logit classifier (LR)	Stratified CV	Rules extracted from DT	DT has recall = 0.80 ± 0.07 and precision = 0.50 ± 0.11 on the injury class, 0.76 AUC	Time since previous injury, HSR variation, total distance monotony
Rossi et al. (2023) [33]	Injury prevention in football	GPS device (K-GPS 10 Hz, K-Sport International, Italy). Coulter blood counter Model S-plus II, VIDAS testosterone (Ref. 30418) and VIDAS cortisol S	External load and blood measurements Injury outcome/definition: The injury label was defined as training sessions occurring within the 7 days preceding a non-contact injury that resulted in absence from physical activity for at least one day after onset.	18 male players from the Italian league (Serie B), seasons 2017/2018 and 2018/2019, 128 sessions per player, 89 blood samples, 3.3 per player	ADASYN, ACWR, RFECV, k-means	DT and XGB	70% training, 30% testing	SHAP	XGB F1-score of the injury class = 63%. Blood sample profiles increase the injury prediction ability by about 15% for the injury class = 63%	Hematocrit, hemoglobin, RBC, ferritin, testosterone, external load (GPS)
Lyubovsky et al. (2022) [37]	Injury prevention in American football	GPS Catapult’s Playertek/physiological sensors (omegawave)	Player stats, training load data, survey, GPS, and omegawave data Injury outcome/definition: All injuries were defined as any condition or complication resulting in partial or complete absence from training sessions or inability to participate in games.	113 days in their 2019 season/during the season, there was a total of 173 recorded injuries from 101 male players	LR	LR, SVM, DT, k-NNs algorithm, and a Gaussian NB	5-fold CV/(one game was out for testing)	NA	Precision of 0.47, recall of 0.74, and an F1-score of 0.52	Previous injury, soreness, wellness/recovery scores
Carey et al. (2018) [38]	Injury prevention in Australian football	10 Hz GPS, 100 Hz triaxial accelerometers	External loads/session RPE/player load Injury outcome/definition: all injuries	Professional Australian football club 2014, 2015 and 2016 seasons, providing 133 player seasons from 75 unique male athletes	PCA	Regularised LR, RF, GEE, and SVM	10-fold CV and 2016 season (hold-out validation)	NA	LR AUC = 0.76	Cumulative load, acute/chronic load, ACWR, monotony, strain

Notes. EPTS = electronic performance and tracking system; AUC = area under the curve; CV = cross-validation; DT = decision tree; GPS = global positioning system; HR = heart rate; IMU = inertial measurement unit; LR = logistic regression; k-NNs = k-nearest neighbours ML = machine learning; NA = not reported; RF = random forest; RNN = recurrent neural network; LSTM = long short-term memory; MLP = multilayer perceptron; SHAP = SHapley Additive exPlanations; SVM = support vector machine; GBDT = gradient boosting decision tree; CART = classification and regression tree; GEE = generalised estimating equation, HRVM = heart rate variability monitor; mRMR = maximum relevance–minimum redundancy.

## 4. Discussion

This scoping review synthesised evidence from 11 studies examining the use of AI and sensor-based monitoring technologies for injury prediction and risk modelling in team sports. Most of the included studies were published within the last three years, reflecting the growing interest in data-driven approaches to support injury prevention. However, the field remains methodologically fragmented, with substantial variation in injury definitions, monitoring inputs, feature engineering procedures, modelling strategies, and validation frameworks. Such heterogeneity limits meaningful comparison across studies and constrains the translation of findings into applied settings.

Given these inconsistencies, the present scoping review was designed to map and critically synthesise the current literature rather than perform quantitative evidence pooling. Importantly, the findings suggest that the current limitations of AI-based injury prediction in team sports are not solely related to modelling sophistication, but also to inconsistent data collection practices, limited dataset quality, insufficient external validation, and the absence of standardised analytical pipelines. Collectively, these issues restrict the reproducibility, generalisability, and practical applicability of current AI-based injury prediction models.

### 4.1. Important Variables and Feature Engineering

Across the included studies, workload-related variables consistently emerged as the most prominent predictors of injury risk. Frequently reported inputs included total distance covered, high-speed running distance, sprint distance, accelerations and decelerations, session duration, and cumulative workload metrics derived from GPS and inertial sensor data. These variables were typically operationalised as daily, weekly, or multi-week aggregates to reflect both acute and chronic training demands. Historical injury information and indicators of training exposure were also commonly incorporated, consistent with their established role as risk factors in sports injury epidemiology. Several studies further included contextual variables such as playing position, match participation, and competition density to capture situational contributors to injury risk. Evidence from individual studies further highlighted the interaction between workload and injury history. For example, recursive feature selection in one investigation reduced the predictor set to three key variables, time since previous injury, variations in high-speed running, and total distance monotony, demonstrating the combined influence of previous injury status and workload fluctuations on injury risk.

These findings are consistent with earlier non-AI research, which has identified training load, particularly external load metrics, as a key modifiable risk factor for injury, with both excessive loads and rapid load increases associated with elevated injury risk [5,6]. Similarly, ACWR-based models have demonstrated that spikes in workload are strongly associated with injury likelihood, further supporting the importance of workload dynamics rather than absolute values alone [7]. In addition to workload-related metrics, some studies incorporated broader physiological or performance-related features.

The distinction between internal and external load is well supported in previous research, where external load reflects the work performed, while internal load captures the individual physiological and psychological response to that work [17,40]. Moreover, recent frameworks emphasise that combining these two dimensions is essential for accurately understanding adaptation and injury risk [13]. Internal load measures, including heart rate-based variables, ratings of perceived exertion, and subjective assessments of wellness or fatigue, were less consistently applied but were reported in some studies to enhance predictive performance when combined with external workload metrics. However, the contribution of these variables was rarely evaluated systematically, and their inclusion was often driven by data availability rather than formal optimisation procedures. In one study, subjective wellness indicators, particularly perceived soreness and overall recovery scores, showed the highest predictive power, outperforming several GPS-derived workload variables. This finding aligns with previous literature suggesting that internal load indicators, such as heart rate and perceived exertion, can provide valuable insight into athlete fatigue and physiological stress, sometimes exceeding the explanatory value of external load metrics alone [11,16]. Additionally, subjective load measures have been shown to be effective, low-cost tools for capturing psychophysiological responses to training [41]. Nevertheless, these findings do not necessarily indicate that internal load indicators are inherently superior to external load metrics for injury prediction. The observed differences may partly reflect variations in study design, methodological approaches, and the use of workload variables derived from arbitrary or non-standardised measurement units. Another important factor may be the excessive number and diversity of external load variables generated by commercial monitoring systems, which often differ substantially between manufacturers and proprietary software platforms. Importantly, the identification of influential predictors within ML models should not be interpreted as evidence of causal relationships. Most included studies were observational and retrospective in nature, meaning that the reported models primarily capture statistical associations and predictive patterns rather than mechanistic explanations of injury occurrence. Consequently, variables such as external workload metrics may indicate increased injury likelihood without necessarily representing direct causal factors.

Feature engineering strategies varied considerably across investigations. While some studies relied primarily on raw sensor outputs or manufacturer-derived workload metrics, others constructed higher-level features using temporal aggregation, rolling averages, and ratio-based indicators such as ACWRs. Several studies applied explicit feature selection procedures, including correlation-based filtering, univariate statistical screening, and model-based importance ranking. In particular, recursive feature elimination (RFE) was employed in some investigations to iteratively remove less informative predictors and retain a reduced subset of variables that optimised model performance. A smaller number of studies implemented dimensionality reduction techniques, such as principal component analysis (PCA) or regularisation-based methods, to address multicollinearity and manage high-dimensional feature spaces. From a practical perspective, the absence of standardised feature engineering and preprocessing pipelines may substantially limit comparability between clubs, competitions, wearable systems, and commercial software platforms. Differences in aggregation windows, proprietary workload calculations, filtering procedures, and firmware updates may lead to inconsistent input representations, thereby affecting both model reproducibility and transferability across sporting environments. Indicatively, commercial data-processing software associated with wearable sensor systems may generate more than 50 workload-related variables, substantially increasing the complexity of data interpretation and feature selection for analysts and practitioners. Consequently, many newly derived variables or composite metrics may require further validation regarding their predictive value and practical relevance before being incorporated into applied injury prediction frameworks.

Despite these advances, traditional (non-AI) studies have largely relied on predefined workload metrics and simpler aggregation strategies, often without systematic feature optimisation, highlighting a key methodological difference between conventional and machine learning approaches [5,17]. Given the inherently imbalanced nature of injury datasets, with relatively few injury events compared with non-injury observations, some studies adopted data-level strategies to mitigate class imbalance. These included oversampling approaches, most notably the Synthetic Minority Over-sampling Technique (SMOTE), to generate artificial injury cases and improve model sensitivity to rare events. However, the use of SMOTE and related techniques was inconsistent, and few studies systematically compared performance with and without synthetic data augmentation. The length of aggregation windows and the frequency of feature updates differed widely, ranging from short-term daily summaries to multi-week cumulative measures. Nevertheless, only a minority of studies clearly reported the criteria used for feature inclusion, feature elimination, or synthetic data generation, or examined the impact of these procedures on predictive performance. In most cases, feature construction and selection were guided by established workload conventions rather than by systematic, cross-validated optimisation.

Across the 11 included studies, several predictors consistently emerged as the most influential factors in injury risk estimation. The most frequently reported and influential variables were external load metrics, particularly total distance, high-speed running distance, sprint distance, and counts of accelerations and decelerations, which also represent the most commonly shared variables across athlete monitoring systems. Cumulative and temporally aggregated workload indicators, such as rolling averages, monotony indices, and acute-to-chronic workload ratios, were also repeatedly identified as important predictors. Previous injury history was one of the most consistent and influential factors across studies, often appearing among the top-ranked features. Internal load indicators, including perceived soreness, wellness or recovery scores, and heart rate-based measures, were also identified as important predictors in multiple investigations. Additionally, studies incorporating physiological and biochemical markers reported that hematological and hormonal variables such as hematocrit, hemoglobin, red blood cell count, ferritin, and testosterone contributed to improved individualised injury prediction.

Overall, these findings are highly consistent with the broader literature, which suggests that injury risk is multifactorial and best explained through the interaction between workload exposure, previous injury, and individual physiological response to training [5,6]. Τhe absence of standardised and transparently reported pipelines for feature engineering, feature selection, dimensionality reduction, and class imbalance handling represents a major source of methodological variability across studies. Differences in how predictors were derived, filtered, transformed, and balanced limit reproducibility and hinder meaningful comparison of modelling approaches.

### 4.2. Machine Learning Approaches

A wide range of ML models was applied across the included studies, reflecting the exploratory nature of this research field. Commonly reported algorithms included logistic regression, decision trees, random forests, support vector machines, gradient-boosted models, and artificial neural networks. In addition, several studies implemented more advanced deep learning approaches, such as recurrent neural networks (RNNs) and long short-term memory (LSTM) architectures, particularly in attempts to capture temporal patterns in training load and injury risk. Extreme gradient boosting (XGBoost) and other ensemble-based techniques were also employed in some investigations to improve predictive performance. Importantly, algorithm selection was often based on empirical performance comparison rather than on theoretical justification or sport-specific modelling rationale. Tree-based and ensemble methods were frequently favoured because of their flexibility and ability to capture non-linear relationships, although few studies critically evaluated whether such approaches were optimal for the structure and quality of the available data.

Several studies implemented and compared multiple algorithms within the same dataset to identify the most suitable modelling approach, while others focused on a single technique tailored to their data structure or prediction objective. Ensemble and tree-based methods, particularly random forests and gradient-boosted models, were frequently employed because of their capacity to model non-linear relationships and complex interactions among workload, historical, physiological, and contextual predictors. Deep learning approaches were primarily used in studies aiming to model sequential or time-dependent patterns in player monitoring data.

Despite the frequent use of advanced algorithms, model development strategies varied substantially. Some studies adopted relatively simple classification frameworks to distinguish between injured and non-injured observations, whereas others attempted probabilistic risk estimation, short-term injury risk forecasting, or readiness prediction across training cycles or competitive periods. A number of investigations addressed class imbalance through resampling techniques, cost-sensitive learning, or threshold optimisation, although these procedures were not applied consistently and were often insufficiently described.

Although several studies reported improved predictive performance relative to traditional statistical approaches, the magnitude and robustness of these improvements varied widely. In many cases, gains in accuracy or discrimination were modest and dependent on the specific dataset, injury definition, and prediction horizon used. Some studies reported moderate performance levels, while others achieved higher discrimination for specific injury types or short-term risk windows. Importantly, model selection was typically driven by empirical comparison rather than by theoretical or domain-specific considerations, and few studies provided explicit justification for algorithm choice. Hyperparameter tuning procedures and optimisation strategies were also inconsistently reported, limiting reproducibility. Moreover, preprocessing pipelines, missing-data handling procedures, and hyperparameter optimisation strategies were inconsistently reported across studies, limiting reproducibility and increasing the potential risk of methodological bias.

Overall, the diversity of modelling approaches highlights both the flexibility of ML for injury prediction and the absence of consensus regarding optimal analytical strategies. The lack of standardised modelling frameworks, combined with inconsistent reporting of training procedures, feature pipelines, and parameter settings, constrains cross-study comparison and hinders cumulative knowledge building. These findings suggest that future research should move beyond purely exploratory model testing toward more principled and transparent modelling pipelines that incorporate domain knowledge, address data imbalance, and report methodological details in a reproducible manner. Hence, the findings indicate that many current AI-based injury prediction models remain primarily exploratory and context-specific rather than clinically validated systems capable of reliable implementation across sporting environments.

### 4.3. Validation Strategies and Predictive Results

Validation strategies varied considerably across the included studies. Most investigations relied on internal validation procedures, including k-fold cross-validation and random or stratified split-sample testing, whereas external validation using independent datasets was rarely undertaken. Only a small number of studies adopted temporally separated training and testing periods to reflect prospective prediction scenarios or season-based forecasting. This predominance of internal validation raises concerns regarding potential overfitting and optimistic estimates of predictive performance, particularly in studies based on single teams or short observation periods.

Performance was reported using a range of metrics, most commonly accuracy, sensitivity, specificity, and the area under the receiver operating characteristic curve (AUC). Several studies also reported precision, recall, or F1-scores to account for class imbalance. Reported predictive performance ranged from modest to high. Some investigations achieved only limited discrimination, with AUC values close to random classification, particularly when general non-contact injuries were considered. In contrast, higher predictive performance was reported in studies focusing on specific injury types, short-term risk windows, or enriched feature sets, including physiological or wellness-related variables. From a practical perspective, models demonstrating high overall accuracy but limited sensitivity to injury events may have restricted value in applied injury prevention settings, since failure to correctly identify true injury cases substantially reduces clinical usefulness.

Substantial variability in performance was observed across studies and was influenced by differences in injury definitions, prediction horizons, class distributions, and feature engineering strategies. In several cases, relatively high accuracy values were achieved despite limited sensitivity to injury events, reflecting the challenges posed by highly imbalanced datasets and the low incidence of injuries in elite team sports.

The lack of standardised validation frameworks and outcome definitions precluded meaningful comparison of predictive performance across studies and limited the ability to draw robust conclusions regarding model superiority. Furthermore, the infrequent use of external validation raises concerns about the generalisability of proposed models beyond the specific teams, seasons, or competitive contexts in which they were developed.

Importantly, not all forms of heterogeneity observed across studies are equally problematic. While differences in model architecture may reflect legitimate analytical exploration, inconsistencies in injury definitions, feature engineering procedures, and validation strategies directly affect reproducibility, comparability, and the ability to externally validate predictive models across teams and sporting contexts.

Collectively, these findings suggest that many currently proposed AI-based injury prediction models may remain insufficiently robust for reliable implementation in real-world clinical or performance settings. Models developed using small, single-team datasets with limited external validation are particularly vulnerable to overfitting and context-specific bias.

### 4.4. Explainability and Model Transparency

In most studies, explainability was operationalised through relatively simple approaches, such as feature importance rankings, regression coefficients, or tree-based importance measures, rather than through comprehensive XAI frameworks capable of supporting clinically interpretable decision-making at the individual athlete level. Only a minority of studies explicitly addressed model interpretability or incorporated XAI techniques. When explainability was considered, feature importance rankings, regression coefficients, or tree-based importance measures were used to identify influential predictors of injury risk. Across these studies, workload-related variables, particularly cumulative and high-intensity running measures, and previous injury history consistently emerged as key contributors to model predictions.

Nevertheless, most investigations prioritised predictive accuracy over interpretability, and few applied advanced XAI methods such as SHAP values or local explanation frameworks to explore individual-level predictions [42,43]. Moreover, explainability was rarely linked to clinical reasoning or decision-making processes, limiting its practical relevance. Given the clinical, operational, and ethical implications of injury prediction, insufficient attention to model transparency represents a critical shortcoming [44]. Interpretable models are necessary to support practitioner trust, enable interdisciplinary communication between data scientists and clinicians, and ensure that predictive outputs can be meaningfully integrated into training and medical decision-making. Consequently, explainability often remained descriptive rather than functionally integrated into applied decision-support processes, limiting its practical utility in interdisciplinary sport environments.

### 4.5. Geographical Distribution of Studies

The geographical distribution of the included studies was predominantly concentrated in Europe and Australia, with comparatively few investigations conducted in North America or other regions. This pattern likely reflects differences in access to wearable monitoring technologies, institutional support for data-driven performance analysis, and established research collaborations in elite team sport environments. In addition, the dominance of football- and rugby-based studies aligns with the popularity of these sports and the routine use of monitoring systems in professional leagues within these regions.

However, this geographical concentration limits the transferability of findings to sporting contexts characterised by different competition structures, training methodologies, and injury profiles. Regions with less developed monitoring infrastructure or different seasonal formats may exhibit distinct workload patterns and risk factors that are not captured in current models. Broader geographical representation, including studies from diverse sporting systems and resource settings, is therefore necessary to enhance the external validity and global applicability of injury prediction models.

### 4.6. Technology Characteristics and Limitations

Wearable technologies, particularly GPS units and IMUs, constituted the primary data sources across the included studies. These systems enable continuous quantification of external workload and movement characteristics, including distance covered, velocity profiles, and acceleration–deceleration patterns. In recent years, alternative tracking technologies have also emerged, including LPS devices and advanced IMU-based solutions. Importantly, these systems rely on different measurement technologies and signal acquisition principles. For example, GPS devices operate through satellite-based positioning, IMUs rely on inertial sensors and estimate movement-related variables from acceleration data across the three movement axes, and LPS technologies are based on ultra-wideband (UWB) radio-frequency infrastructure. Despite the increasing use of these systems in athlete monitoring, relatively few studies have directly examined the relationships and comparability of variables generated across different tracking technologies. Existing evidence suggests that LPS devices may demonstrate higher validity in open-field environments compared with some GPS devices operating at 10 Hz sampling frequency, although findings remain inconsistent across settings and movement demands. Consequently, further validation studies are required to investigate the relationships, equivalence, and interoperability of workload variables derived from different monitoring systems and reference technologies [45,46,47].

Compared with traditional GPS devices, which rely on satellite signals and are primarily suited for outdoor environments, LPS technologies operate using local radio-frequency infrastructure and can provide higher spatial accuracy and reliability in indoor settings or stadiums with signal obstruction. IMU-based systems, on the other hand, capture high-frequency motion data (e.g., acceleration, angular velocity) and are particularly effective for quantifying rapid changes in movement and mechanical load. Hybrid systems that combine GPS, LPS, and IMU data streams are increasingly used to enhance measurement precision and provide a more comprehensive representation of athlete workload.

Nevertheless, such technologies are subject to several technical constraints. Measurement error, signal loss, and inter-device variability may affect data quality, particularly in environments with limited satellite reception or during high-intensity movements. For example, GPS accuracy may deteriorate during rapid accelerations, changes in direction, or in congested playing environments, whereas LPS devices require infrastructure installation and calibration, which may limit their accessibility. In addition, substantial differences exist in sampling frequency, data filtering procedures, and proprietary algorithms used by manufacturers to derive workload metrics, which may compromise comparability across studies. Furthermore, firmware and software updates introduced by manufacturers over time may alter signal processing, workload calculations, and variable outputs, potentially affecting longitudinal data consistency and comparability across seasons or studies.

Beyond technical issues, reliance on sensor-derived metrics alone may provide an incomplete representation of injury risk. Physiological, psychological, and contextual contributors, such as fatigue, sleep quality, stress, recovery status, and match congestion, are often underrepresented or omitted. The limited integration of multimodal data sources may restrict model sensitivity to complex injury mechanisms. These findings highlight the need for predictive systems that combine wearable-derived variables with internal load, wellness, and contextual data to better reflect the multifactorial nature of sports injuries. Ultimately, there is a growing need for the development of integrated workload indicators capable of simultaneously incorporating external load, internal load, physiological responses, contextual factors, and recovery-related parameters in order to provide a more comprehensive and ecologically valid representation of athlete burden and injury risk.

### 4.7. Limitations of the Included Studies

Several methodological limitations were evident across the included studies. Sample sizes were frequently small and often restricted to single teams or seasons, limiting statistical power and increasing the risk of overfitting. Observation periods were typically short, reducing the ability to capture long-term injury patterns and cumulative workload effects. Inconsistent injury definitions and outcome measures further complicated cross-study comparison, with some investigations focusing on time-loss injuries and others including any physical complaint.

Many studies employed retrospective designs based on historical monitoring and injury records, which may introduce bias related to data completeness, reporting accuracy, and temporal alignment between predictors and outcomes. Handling of missing data was seldom described in detail, and few studies reported sensitivity analyses to assess the robustness of their findings. Moreover, most investigations focused on model development and performance metrics, while relatively little attention was paid to clinical interpretability, implementation feasibility, or the potential behavioural consequences of predictive outputs. The lack of prospective validation and real-world testing remains a substantial barrier to translation.

### 4.8. Limitations of This Scoping Review

This scoping review has several limitations that should be acknowledged. The search strategy was restricted to studies published in English and indexed in PubMed and Scopus, which may have resulted in the omission of relevant research reported in other languages or databases. Grey literature and unpublished studies were not included, potentially introducing publication bias. The exclusion of grey literature may also have limited the inclusion of emerging methodological developments in this rapidly evolving field, particularly given the pace of innovation in wearable technologies and applied ML. The substantial heterogeneity in study designs, injury definitions, and analytical approaches precluded quantitative synthesis or formal meta-analysis. Therefore, conclusions regarding the relative validity, robustness, or superiority of specific modelling approaches should be interpreted cautiously.

In addition, as a scoping review, the primary objective was to map the extent and nature of existing research rather than to evaluate methodological quality or risk of bias. Consequently, no formal critical appraisal of included studies was undertaken. Although this approach is consistent with scoping review methodology, it limits the ability to draw definitive conclusions regarding the relative effectiveness of different modelling approaches.

Finally, a quantitative meta-analysis was not undertaken because of the substantial methodological and clinical heterogeneity across the included studies. Differences in injury definitions, prediction outcomes, monitoring systems, feature engineering procedures, machine learning architectures, validation strategies, and performance reporting precluded meaningful statistical pooling and direct quantitative comparison.

### 4.9. Future Research Directions

Future research should prioritise the development of standardised injury definitions and harmonised feature engineering procedures to improve comparability and reproducibility across studies. Greater emphasis should be placed on robust validation frameworks, including external validation across multiple teams, seasons, and competitive levels. Prospective study designs are required to assess predictive performance in real-world conditions and to determine whether model-informed interventions can meaningfully reduce injury incidence.

Importantly, future models should be trained on multi-team and multi-season datasets and externally tested on independent cohorts, in order to improve generalisability and reduce overfitting to team-specific characteristics. Current evidence suggests that many models are developed using single-team datasets, which limits their applicability across different sporting environments. This issue has also been highlighted in previous literature on training load and injury risk, where findings are often context-specific and influenced by team structure, competition demands, and monitoring practices [5,6].

In addition, future research should extend beyond a narrow focus on football and rugby and examine the applicability of injury prediction models across a broader range of team sports, including basketball, volleyball, futsal, and handball. Differences in game structure, physical demands, and scheduling may influence both workload patterns and injury mechanisms, and therefore model performance. Expanding research across sports contexts is essential to improve the external validity and practical applicability of AI-based injury prediction systems, as also suggested in athlete monitoring and tracking literature [14].

Advances in ΧAΙ should be leveraged to enhance transparency and interpretability, enabling practitioners to understand which variables drive risk predictions and why. Integration of physiological, psychological, and contextual variables alongside sensor-derived data may improve predictive accuracy and clinical relevance. Additionally, future work should explore ethical considerations, including data privacy, informed consent, and the potential unintended consequences of algorithm-driven decision-making in sport.

### 4.10. Practical Implications and Guidelines

From an applied perspective, injury prediction models should be implemented as decision-support tools rather than as deterministic or prescriptive systems. Predictive outputs should be interpreted alongside clinical assessment, coaching insight, and contextual information such as competition schedule and athlete readiness. Overreliance on algorithmic predictions without domain expertise may increase the risk of inappropriate training modifications or misclassification of injury risk.

To enhance real-world applicability, practitioners and organisations should prioritise the use of models that have been validated across multiple teams and, where possible, across different sports contexts, rather than relying on models developed within a single-team environment. Models trained and tested on diverse populations are more likely to capture the variability in training practices, competition demands, and athlete characteristics that exist across teams and sports.

Transparent reporting of model inputs, assumptions, and limitations is essential to avoid misinterpretation and to promote responsible use. Practitioners should be trained to understand basic principles of model uncertainty and performance metrics. The development of interdisciplinary teams involving sports scientists, clinicians, and data scientists is strongly recommended to ensure that predictive systems are technically robust, clinically meaningful, and ethically sound. Clear operational protocols should be established to define how predictive information is communicated and acted upon within performance and medical teams.

## 5. Conclusions

This scoping review mapped current applications of AI and sensor-based monitoring technologies for injury prediction in team sports. Across the included studies, injury risk was most consistently associated with three groups of predictors: external workload metrics, previous injury history, and internal or physiological indicators of recovery and readiness. These findings reinforce the multifactorial nature of injury risk and highlight the importance of integrated athlete monitoring approaches.

However, substantial heterogeneity was observed in data sources, feature engineering procedures, modelling strategies, and validation frameworks. Most studies relied on single-team datasets and internal validation, with limited use of external validation, multimodal data integration, or explainable modelling approaches. These limitations restrict the generalisability and practical applicability of current predictive models.

In an era characterised by the rapid expansion of athlete monitoring data, there is a growing need for the development of integrated monitoring ecosystems in which data collection, processing, and analytical methodologies are standardised across indoor and outdoor team sports. Validation studies examining the accuracy and comparability of workload monitoring systems, together with the reduction in and optimisation of monitoring variables representing specific components of internal and external load, may facilitate the long-term identification of the parameters most strongly associated with injury risk prediction.

AI-based injury prediction systems should therefore be considered decision-support tools rather than deterministic predictors. Future research should prioritise methodological standardisation, the integration of multiple data sources, and robust external validation across teams and contexts. Addressing these challenges is essential for developing reliable, interpretable, and clinically meaningful AI-driven tools that can effectively support injury prevention in team sports.

## Data Availability

No new data were created or analyzed in this study. Data sharing is not applicable to this article.

## Supplementary Materials

The following supporting information can be downloaded at: https://www.mdpi.com/article/10.3390/jfmk11020204/s1, Supplementary File S1: Search Strategy.

## Appendix A

**Table A1:** Eligibility criteria according to an adapted PICOS framework.

PICOS Element	Description
Population	Team sport athletes
Intervention/exposure	AI/ML-based injury prediction using wearable/sensor monitoring
Comparator	Not mandatory
Outcomes	Injury occurrence, injury risk, injury-related outcomes
Study design	Peer-reviewed original studies
