# Supplementary material for: Injury Prediction and Risk Modelling in Team Sports Using Artificial Intelligence and Sensor-Based Monitoring: A Scoping Review
Source: J Funct Morphol Kinesiol. 2026 May 22;11(2):204. doi: 10.3390/jfmk11020204 (PMC13262898; doi:10.3390/jfmk11020204)
Supplement: Supplementary file 1 [file jfmk-11-00204-s001.zip › Supplementary File S1. Search Strategy.pdf]

Supplementary File S1. Search Strategy.

Pubmed

(

soccer[tiab] OR football[tiab] OR "association football"[tiab]

OR basketball[tiab] OR "basket ball"[tiab]

OR rugby[tiab] OR "rugby union"[tiab] OR "rugby league"[tiab]

OR handball[tiab] OR "team handball"[tiab]

OR volleyball[tiab] OR futsal[tiab]

OR hockey[tiab] OR "ice hockey"[tiab] OR "field hockey"[tiab]

OR "gaelic football"[tiab]

OR "australian football"[tiab] OR "aussie rules"[tiab]

OR "american football"[tiab]

OR "team sport*"[tiab]

)

AND

(

injury[tiab] OR injuries[tiab]

OR "musculoskeletal injur*"[tiab]

OR "soft tissue injur*"[tiab]

OR hamstring[tiab]

OR "anterior cruciate"[tiab] OR ACL[tiab]

OR sprain*[tiab] OR fracture*[tiab]

OR tendinopathy[tiab]

OR "non-contact injur*"[tiab]

OR "acute injur*"[tiab] OR "chronic injur*"[tiab]

```
  OR "traumatic injur*"[tiab] OR "contact injur*"[tiab]
  OR "recurrent injur*"[tiab] OR exacerbation[tiab]
  OR "time-loss injur*"[tiab]
  OR "muscle strain"[tiab] OR "pulled muscle"[tiab]
  OR "muscle tear"[tiab] OR "muscle rupture"[tiab]
  OR "muscle contusion"[tiab] OR "muscle spasm"[tiab]
  OR tendinitis[tiab] OR tendonitis[tiab] OR tendinosis[tiab]
  OR "tendon rupture"[tiab]
  OR "Achilles tendinopathy"[tiab] OR "patellar tendinopathy"[tiab]
  OR dislocation*[tiab] OR subluxation*[tiab]
  OR "joint instability"[tiab] OR "joint effusion"[tiab]
  OR "meniscus tear"[tiab] OR "labral tear"[tiab]
  OR "cartilage injur*"[tiab] OR "osteochondral defect*"[tiab]
  OR "stress fracture*"[tiab] OR "bone contusion"[tiab]
  OR sciatica[tiab] OR neuropathy[tiab] OR "nerve injur*"[tiab]
  OR pain[tiab] OR bursitis[tiab] OR fasciitis[tiab]
  OR "compartment syndrome"[tiab] OR "shin splints"[tiab]
  OR "low back pain"[tiab]
)
AND
(
  prognosis[tiab] OR prognos*[tiab] OR predict*[tiab]
  OR "risk factor*"[tiab] OR "early detection"[tiab]
  OR forecast*[tiab] OR screening[tiab]
  OR "injury incidence"[tiab]
  OR "injury prevalence"[tiab]
```

```
  OR "injury burden"[tiab]
  OR exposure[tiab]
  OR severity[tiab]
  OR "days lost"[tiab]
  OR "recurrence rate"[tiab]
)
AND
(
  "artificial intelligence"[tiab] OR AI[tiab]
  OR "machine learning"[tiab] OR ML[tiab]
  OR "deep learning"[tiab] OR DL[tiab]
  OR "neural network*"[tiab]
  OR "data mining"[tiab]
  OR "AI-assisted monitoring"[tiab]
  OR "predictive analytics"[tiab]
  OR "decision-support system*"[tiab]
)
AND
(
  GPS[tiab] OR GNSS[tiab] OR "global positioning system"[tiab]
  OR "Global Navigation Satellite System"[tiab]
  OR IMU[tiab] OR IMUs[tiab]
  OR "inertial measurement unit*"[tiab]
  OR "wearable sensor*"[tiab] OR acceleromet*[tiab]
  OR gyroscop*[tiab] OR microtechnology[tiab]
  OR "performance monitoring"[tiab]
```

OR "in-game monitoring"[tiab]

OR "player monitoring"[tiab]

OR "athlete tracking"[tiab]

OR "wearable technology"[tiab]

OR "smart sensor*"[tiab]

OR "digital performance monitoring"[tiab]

OR LPS[tiab] OR "Local Positioning System"[tiab]

OR magnetometer[tiab]

OR UWB[tiab] OR "Ultra-Wideband"[tiab]

OR RFID[tiab]

OR "heart rate telemetry"[tiab]

OR "smart vest"[tiab]

OR "training load"[tiab]

OR "acute load"[tiab] OR "chronic load"[tiab]

OR "acute chronic workload ratio"[tiab] OR ACWR[tiab]

OR "cumulative load"[tiab]

OR "fatigue monitoring"[tiab]

OR readiness[tiab] OR "recovery status"[tiab]

OR "overtraining risk"[tiab]

OR "injury risk profiling"[tiab]

OR "monitoring of game intensity"[tiab]

OR "match demands"[tiab]

OR "work-rate analysis"[tiab]

OR "performance profiling"[tiab]

)

Scopus

```
TITLE-ABS-KEY (
    soccer OR football OR "association football"
    OR basketball OR "basket ball"
    OR rugby OR "rugby union" OR "rugby league"
    OR handball OR "team handball"
    OR volleyball OR futsal
    OR hockey OR "ice hockey" OR "field hockey"
    OR "gaelic football"
    OR "australian football" OR "aussie rules"
    OR "american football"
    OR "team sport*"
)
AND
TITLE-ABS-KEY (
    injury OR injuries OR "musculoskeletal injury*"
    OR "soft tissue injury*" OR hamstring
    OR "anterior cruciate" OR ACL
    OR sprain* OR fracture* OR tendinopathy
    OR "non-contact injur*" OR "acute injur*" OR "chronic injur*"
    OR "traumatic injur*" OR "contact injur*" OR "recurrent injur*"
    OR exacerbation OR "time-loss injur*"
    OR "muscle strain" OR "pulled muscle"
    OR "muscle tear" OR "muscle rupture"
```

```
    OR "muscle contusion" OR "muscle spasm"
    OR tendinitis OR tendonitis OR tendinosis
    OR "tendon rupture"
    OR "Achilles tendinopathy" OR "patellar tendinopathy"
    OR dislocation* OR subluxation*
    OR "joint instability" OR "joint effusion"
    OR "meniscus tear" OR "labral tear"
    OR "cartilage injur*" OR "osteochondral defect*"
    OR "stress fracture*" OR "bone contusion"
    OR sciatica OR neuropathy OR "nerve injur*"
    OR pain OR bursitis OR fasciitis
    OR "compartment syndrome" OR "shin splints"
    OR "low back pain"
)
AND
TITLE-ABS-KEY (
    prognosis OR prognos* OR predict*
    OR "risk factor*" OR "early detection"
    OR forecast* OR screening
    OR "injury incidence" OR "injury prevalence"
    OR "injury burden" OR exposure
    OR severity OR "days lost" OR "recurrence rate"
)
AND
TITLE-ABS-KEY (
    "artificial intelligence" OR AI
```

```
    OR "machine learning" OR ML
    OR "deep learning" OR DL
    OR "neural network*" OR "data mining"
    OR "AI-assisted monitoring"
    OR "predictive analytics"
    OR "decision-support system*"
)
AND
TITLE-ABS-KEY (
    GPS OR GNSS OR "global positioning system"
    OR "Global Navigation Satellite System"
    OR IMU OR IMUs OR "inertial measurement unit*"
    OR "wearable sensor*" OR acceleromet*
    OR gyroscop* OR microtechnology
    OR "performance monitoring"
    OR "in-game monitoring"
    OR "player monitoring"
    OR "athlete tracking"
    OR "wearable technology"
    OR "smart sensor*"
    OR "digital performance monitoring"
    OR LPS OR "Local Positioning System"
    OR magnetometer
    OR UWB OR "Ultra-Wideband"
    OR RFID
    OR "heart rate telemetry"
```

OR "smart vest"

OR "training load"

OR "acute load" OR "chronic load"

OR "acute chronic workload ratio" OR ACWR

OR "cumulative load"

OR "fatigue monitoring"

OR readiness OR "recovery status"

OR "overtraining risk"

OR "injury risk profiling"

OR "monitoring of game intensity"

OR "match demands"

OR "work-rate analysis"

OR "performance profiling"

)
